# Creating Awareness for Primary Immunodeficiencies in Japan

**DOI:** 10.3389/fimmu.2021.803459

**Published:** 2021-12-13

**Authors:** Hidetoshi Takada

**Affiliations:** Department of Child Health, Faculty of Medicine, University of Tsukuba, Tsukuba, Japan

**Keywords:** primary immunodeficiency, inborn errors in immunity, nationwide survey, interleukin-1 receptor-associated kinase 4 (IRAK-4), questionnaire research

## Abstract

Primary immunodeficiency (PID) is primarily characterized by susceptibility to infectious diseases. In addition, patients with some type of PID are prone to develop autoimmune, autoinflammatory, or malignant diseases. Therefore, the term, inborn errors of immunity (IEI), has been more used rather than PID. In recent years, the number of diseases which belong to PID has been increasing. There were approximately 110 diseases in the report of International Union of Immunological Societies in 1999. Since then, the number increased to 430 diseases in the latest IUIS report in 2019. We conducted PID nationwide survey in Japan for 3 times in the last 15 years. These studies were focused on incidence and complications of PID, the clinical course of viral infection, and methods to prevent infectious diseases in PID patients. For the awareness of PID, it is essential to know the general and fundamental information of PID patients. Needless to say, we need it to offer appropriate medical services for PID patients. Moreover, chances to provide answers to the questionnaires and seeing the results of the analysis should contribute to the awareness of PID among doctors. In this review, I am going to summarize the results of 3 nationwide survey in Japan, and pick up interleukin-1 receptor-associated kinase 4 (IRAK4) deficiency as an example for creating awareness for its appropriate management.

## Nationwide Survey of PID Patients in Japan 2007

We conducted a nationwide questionnaire survey in 2007 to clarify the incidence and the clinical characteristics of PID in Japan (1). The study was conducted according to the nationwide epidemiological survey manual of patients with intractable disease (2nd edition 2006, Ministry of Health, Labour, and Welfare of Japan) (2). Questionnaires were sent to 1224 pediatric departments and 1670 internal medicine departments of hospitals. Primary questionnaire was aimed to reveal the prevalence of PID in Japan, and the clinical data of the patients were obtained by the secondary questionnaire. The response rates for the questionnaire survey from the pediatric departments and the departments of internal medicine were 55.5% and 20.1%, respectively. We found that the number of PID patients was estimated to be 2900 (95% confidence interval: 2300-3500), and the prevalence of PID in Japan was 2.3/100,000 inhabitants. The prevalence was equivalent to that reported from Singapore (3) and Taiwan (4) (2.7/100,000 and 0.8-2.2/100,000, respectively), but was lower than that reported from Europe, 4.4/100,000 in France and 6.8 in Norway (5, 6).

By the secondary questionnaire, we found that predominantly antibody deficiency was the most common (40%), followed by congenital defects of phagocyte number, function or both (19%) among the PID disease categories. Bruton’s tyrosine kinase (BTK) deficiency was found to be the most common PID in Japan, and accounted for a higher rate (14.7%) than in European countries (5.9%) (7). On the other hand, the prevalence of common variable immunodeficiency (CVID) was low (11.0%). One of the reasons why the prevalence of BTK deficiency was high and that of common variable immunodeficiency (CVID) was low in this study compared with that reported from Europe might be the high questionnaire response rate from pediatric departments compared with that from department of internal medicine in this study. The prevalence of chronic granulomatous disease (CGD) (11.9%) was also found to be the common PID in Japan. Although the genetic background of Japanese CGD is different from that of European countries, the prevalence seems to be similar (8).

This study also revealed that malignant diseases were observed in 2.7% of PID patients, as previously published (1). Epstein–Barr virus-related or unrelated lymphoma, and leukemia were found predominantly. CVID, Wiskott–Aldrich syndrome, and ataxia telangiectasia were frequently associated with such malignant diseases with the percentages of 7.0%, 8.1% and 1.3%, respectively. Complication of immune-related diseases were also found in 8.5% of PID patients, especially in patients with autoimmune lymphoproliferative syndrome and immune dysregulation, polyendocrinopathy, enteropathy X-linked. Interestingly, they were also observed in nuclear factor kappa B (NF-κB) essential modulator (NEMO) deficiency patients. The main immune-related complication was immune related bowel disease, such as inflammatory bowel disease and autoimmune enteritis, which was found in 3.6% of PID patients. Other unique complications of PID patients were immune related and non-immune-related endocrine diseases (9). Some of these complications were clearly associated with the genetic cause of PID, but others were difficult to explain the pathogenesis in connection with it. The most common endocrine complication was hypoparathyroidism which was found in1.6% of total PID patients. Fourteen out of 15 PID patients complicated with hypoparathyroidism had DiGeorge syndrome. The other patient had autoimmune polyendocrinopathy-candidiasis-ectodermal syndrome (APECED). Non-autoimmune hypothyroidism, autoimmune hypothyroidism, type 1 diabetes were found in 0.8%, 0.5% and 0.9%, respectively. The incidence of such endocrine complications in PID patients was higher than that in general population.

## Nationwide Survey of PID Patients in Japan 2013

The severity and clinical course of common viral infections in PID patients had not been investigated in a large cohort of PID patients. We conducted the nationwide questionnaire study in 2013 to reveal the clinical course of respiratory syncytial (RS) virus, rotavirus, influenza virus, and varicella-zoster virus infections in PID patients (10). These 4 infectious diseases were chosen because the pathogens are usually identified with sufficient accuracy by using rapid antigen detection test (RS virus, rotavirus and influenza virus) or the correct diagnosis can be made by clinical manifestation (varicella). We found that 51 of 910 (5.6%) PID patients were hospitalized for the treatment of one of the 4 viral infectious diseases. Patients with cellular immunodeficiency (combined immunodeficiency and combined immunodeficiencies with associated or syndromic features) shared 32.7% of total PID patients. As expected, the ratios of patients with cellular immunodeficiency among hospitalized PID patients for the treatment of RS virus, Rotavirus, influenza and varicella-zoster virus infection were 53.3%, 40.0%, 35.0% and 60.0%, respectively. Patients with cellular immunodeficiency tended to have a longer duration of hospital stay, increased ratio of needs for mechanical ventilation, and/or death from these infectious diseases (10). These findings support the pivotal role of cellular immunity against these viral infections, and warrant the application of palivizumab to PID patients, with cellular immunodeficiency, in particular.

## Nationwide Survey of PID Patients in Japan 2018

We conducted an additional nationwide survey in Japan in 2018, focusing on protective measures for PID patients against infectious diseases (11). In this study, a total of 1,307 patients were reported. Therefore, the prevalence of PID was estimated to be 2.2 patients per 100,000 population, which was similar to the result of nationwide survey of 2007. The most common disease category was autoinflammatory disorders (25%), followed by antibody deficiencies (24%) and congenital defects of phagocyte number or function (16%) It was surprising because the most common disease category was predominantly antibody deficiency, followed by congenital defects of phagocyte number, function or both in the 2007 survey. Among autoinflammatory syndrome, the most common disease was familial Mediterranean fever. The increased ratio of familial Mediterranean fever might be due to the spreading knowledge of familial Mediterranean fever, which had previously been believed to be rare in Japan (12).

In this study, we first focused on the vaccination for the PID patients. One of the crucial findings was that a significant number of patients received contraindicated vaccines (12). Severe combined immunodeficiency (SCID) is not routinely screened at birth in Japan. These vaccines were administered mainly because the patients were not diagnosed as PID by the time of vaccination. Actually, forty-three percent of PID patients for whom bacillus Calmette-Guérin (BCG) vaccination was contraindicated were inoculated with BCG, and 14% of the inoculated patients developed BCG infections. BCG infections developed predominantly in patients with CGD and Mendelian susceptibility to mycobacterial diseases (MSMD). BCG is scheduled for children during the first year of life in Japan, and Japan Pediatric Society recommends BCG inoculation between 5 and 7 months after birth. The method to diagnose very early in life before the vaccination age should be established for patients with CGD and MSMD in addition to the SCID patients. Other statistical analysis regarding methods of prevention against infectious diseases is ongoing.

## Prevention of Invasive Bacterial Infection in Patients With Interleukin-1 Receptor-Associated Kinase 4 Deficiency

Among PID which is comprised of more than 430 diseases, IRAK4 deficiency is one of the PID to which we should create awareness. We have compiled data of all Japanese IRAK4 deficient patients independent of the nationwide studies. IRAK4 deficiency patients are at increased risk of having invasive bacterial infection, predominantly caused by pneumococcus, *Staphylococcus aureus*, streptococci, and *Pseudomonas aeruginosa*. Especially, pneumococcal meningitis is the most common serious infection in Japanese patients (13). The disease cannot be diagnosed unless the doctors have an awareness of the disease, because it lacks the specific laboratory findings. IRAK4 is a molecule, similar to myeloid differentiation primary response gene 88 (MyD88), which mediates signal transduction from most Toll-like receptors (TLR), IL-1 receptor, and IL18 receptor. The clinical manifestation of MyD88 deficiency is very similar to that of IRAK4 deficiency. For these diseases, the establishment of preventive method against invasive bacterial infection is of extreme importance, because the mortality rate of the invasive bacterial infection is as high as approximately 50%, and intriguingly, the patients become less susceptible to these infectious pathogens after infancy or early childhood. We investigated serotype-specific opsonophagocytic activity in serum from IRAK4 deficient patients before and after the vaccination of conjugated or non-conjugated pneumococcal vaccine to determine whether anti-pneumococcal vaccination was effective or not (14). We found that IRAK4 deficiency patients produced protective levels of anti-pneumococcal antibody after the vaccination. Therefore, pneumococcal vaccination schedules are recommended.

On the other hand, pneumococcal vaccination cannot cover all pneumococcal serotypes. Conjugated vaccine contains only 13, and non-conjugated vaccine contains 23 polysaccharide antigens. Invasive pneumococcal infection caused by non-vaccine strain is increasing after the application of conjugated pneumococcal vaccine in Japan (15, 16). We therefore recommend the use of penicillin and sulfamethoxazole-trimethoprim at least until 8 years of age, in addition to the conjugated vaccine followed by non-conjugated pneumococcal vaccine for the prophylaxis of invasive pneumococcal infection (14). Moreover, especially during the infantile period and early childhood, regular immunoglobulin therapy may be required. This strategy to prevent invasive bacterial infection should also be applied to MyD88 deficiency.

## Summary

General and fundamental information is necessary for the appropriate diagnosis and management of PID patients. Genetic diagnosis is essential for the diagnosis of PID. We have to take into account any difference of clinical manifestation based on the genetic background or ethnic groups, which was typically found in familial Mediterranean fever. Nationwide study and national registration system may further contribute to the accumulation of information how to assess PID patients. It has been reported that neonatal screening of SCID plays an important role for the early diagnosis and fair outcome after hematopoietic stem cell transplantation. We are expecting the establishment of screening system for patients with CGD and MSMD, because the patients might receive BCG which cause severe and life-threatening BCG infection in Japan. PID is a rare disease. To offer best medical services to PID patients, we have to offer an easy access to updated information regarding PID for doctors.

In conclusion, recent nationwide questionnaire surveys which were carried out 3 times in the last 15 years clarified the prevalence and clinical characteristics of PID. And the importance of nationwide study of specific disorders was shown by considering IRAK4 deficiency as an example. Furthermore, we clarified complications of PID, problems of vaccination in PID patients and needs for the newborn screening.

## Author Contributions

The author confirms being the sole contributor of this work and has approved it for publication.

## Conflict of Interest

The author declares that the research was conducted in the absence of any commercial or financial relationships that could be construed as a potential conflict of interest.

## Publisher’s Note

All claims expressed in this article are solely those of the authors and do not necessarily represent those of their affiliated organizations, or those of the publisher, the editors and the reviewers. Any product that may be evaluated in this article, or claim that may be made by its manufacturer, is not guaranteed or endorsed by the publisher.
